# Reconstruction of a Nasal Defect With a Radial Forearm Flap Following Trauma of a Paramedian Forehead Flap

**Published:** 2017-06-29

**Authors:** Benson J. Pulikkottil, Ronnie A. Pezeshk, James F. Thornton, Nicholas T. Haddock

**Affiliations:** Department of Plastic and Reconstructive Surgery, University of Texas Southwestern Medical Center, Dallas

**Keywords:** paramedian forehead flap, radial forearm arm free flap, microsurgery, nasal reconstruction, Mohs surgery

## DESCRIPTION

**Figure 1:**
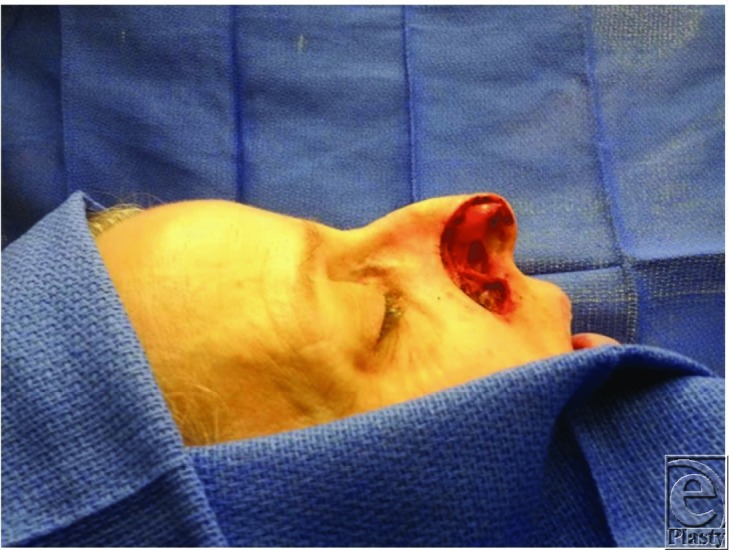
3-cm nasal wound after Mohs surgery for basal cell carcinoma of the right ala.

**Figure 2:**
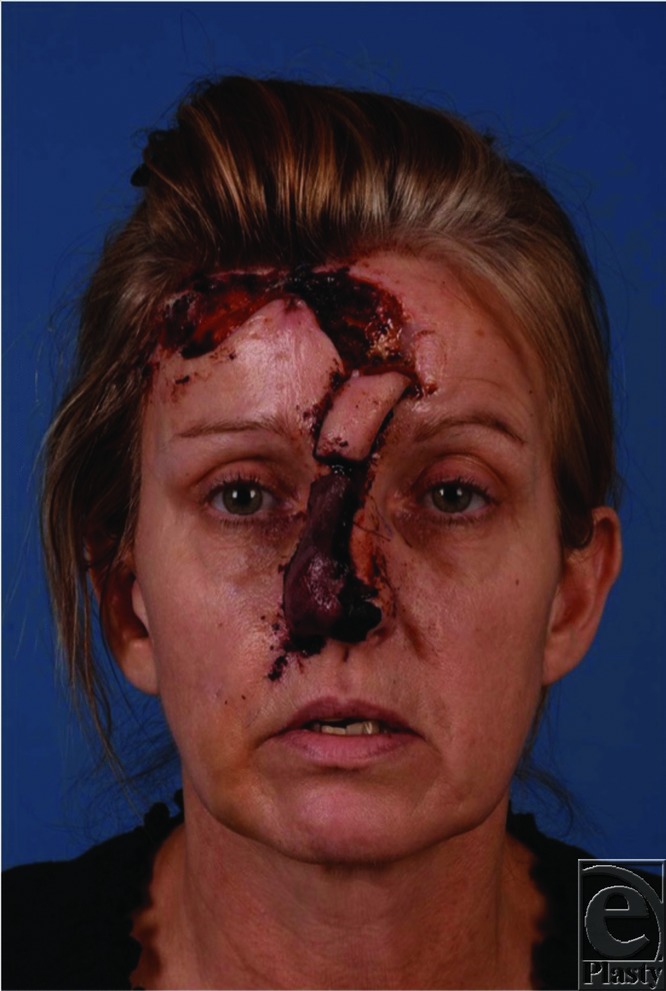
Post-operative day 7 as patient presents to clinic after flap was unintentionally divided by home care nursing staff.

**Figure 3:**
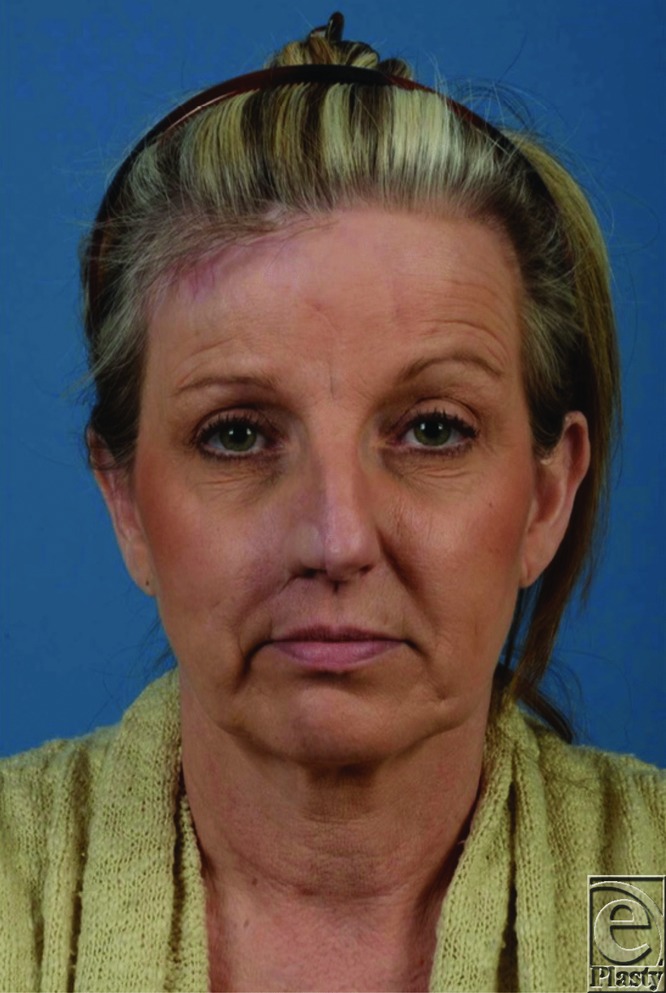
Status post radial forearm free flap reconstruction to repair nasal defect.

**Figure 4:**
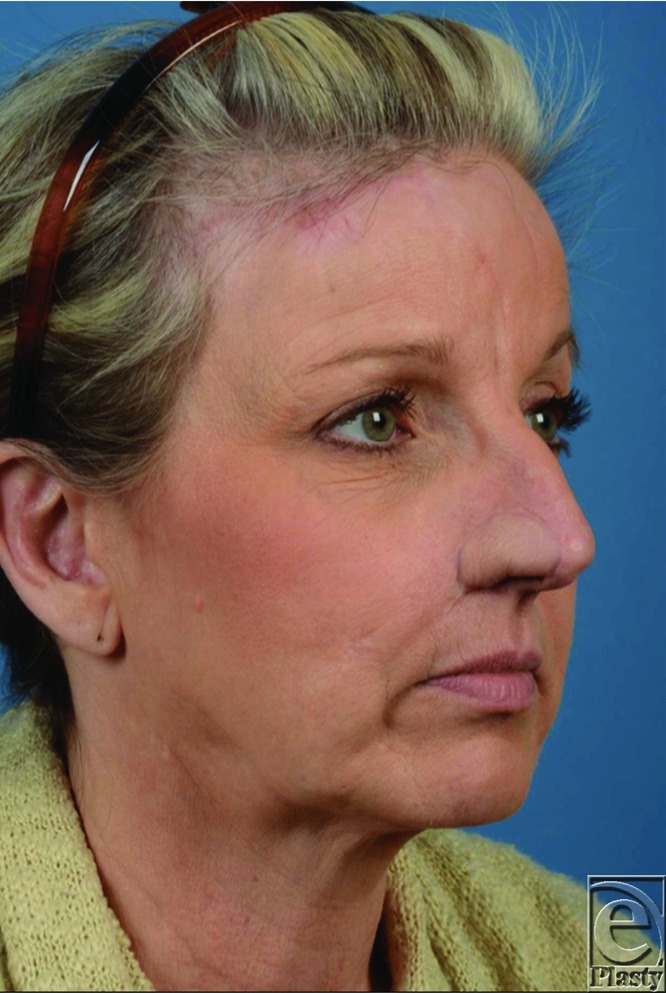
Status post radial forearm free flap reconstruction to repair nasal defect.

A 49-year-old woman presented with a 3-cm nasal wound after Mohs surgery for basal cell carcinoma of the right ala (Fig 1). She underwent reconstruction with a cervicofacial advancement flap closure, left paramedian forehead flap with turn in for lining, and left mucosal soft tissue advancement, as well as a right ear conchal cartilage graft for the right ala. On postoperative day 7, she presented after the flap was unintentionally divided by the home care nursing staff during a dressing change (Fig 2). The reconstructed portion was nonviable. She successfully underwent a radial forearm free flap reconstruction to repair this defect (Figs 3 and 4).

## QUESTIONS

1. **What should be discussed with patients before performing nasal reconstruction?**
2. **What prevented the team from performing a second paramedian forehead flap reconstruction after the inadvertent division?**
3. **Why was the radial forearm flap a viable alternative in this situation?**
4. **How can we better address communication with nursing staff regarding postoperative care?**

## DISCUSSION

Nasal reconstruction is the most common, yet arduous, procedure after Mohs surgery. Our eyes see the unexpected and disregard what is or simulates normal. Flaps are designed to replace topographic units, not merely to conceal defects.^1^ Patients who seek nasal reconstruction are generally referred to the plastic surgeon and will appreciate an in-depth explanation of the road that lies ahead for them. A thorough preoperative plan must include a comprehensive evaluation as well as a discussion establishing expectations and determining the patient's tolerance for a single- or multiple-stage procedure, if necessary. All risks, benefits, and alternatives to the procedure must be explained in detail. Allowing the patients to actively engage in their preoperative plan will not only put them at ease but also prepare them for any unforeseen circumstances that may occur.

In a patient suffering from a heminasal defect, the paramedian forehead flap is considered the gold standard of treatment.^2^ There is no other flap that can precisely match both color and texture and simulate what we perceive to be normal. This is a reliable and versatile flap that possesses a robust vascular supply, rich in collaterals based primarily on the supratrochlear artery and supraorbital plexus.^3^ The flap is generally designed contralateral to the defect to allow minimal kinking at the pivot point.^4^ The surgeon must be prepared to deal with complications such as poor donor site healing, flap necrosis, hematoma formation, impaired nasal function, as well as a poor aesthetic outcome.^1^ In this case, performing an ipsilateral paramedian forehead flap reconstruction would have allowed us to perform a contralateral flap reconstruction after the initial reconstruction flap was divided. Performing a contralateral flap reconstruction damaged critical blood vessels necessary for a subsequent ipsilateral flap.

In cases where a paramedian forehead flap is not appropriate due to size, size, depth of tissue loss, or irradiation, a radial forearm free flap can be successfully employed with good aesthetic results.^5^ This flap provides a suitable thickness and width for both resurfacing the nasal surface and lining without notable contraction intranasally, allowing the airway to be maintained. There is also no excessive scarring of the donor site on the face.

There has been a successful partnership with our visiting nurses who have provided outstanding care to our patients for many years. Despite this, we have been forced to examine how we communicate with home care services once our patients are discharged from the hospital. This is particularly evident with new nurses who lack the experience of their seasoned colleagues or ones who have not cared for this patient population in the past. Protocols must be established in the form of an in-service training for nursing staff that accurately describes the procedure as well as what to expect during all phases of the postoperative period. Spending more time to educate the patient as well as providers outside the hospital setting can help prevent situations such as this in the future.
